# Maternal Severity and Diagnostic Validity of the Revised Japanese Criteria for Obstetric Disseminated Intravascular Coagulation: A Retrospective Observational Study

**DOI:** 10.1111/jog.70388

**Published:** 2026-06-30

**Authors:** Yutaro Takahashi, Soichiro Obata, Sayuri Nakanishi, Ryosuke Shindo, Shigeru Aoki, Etsuko Miyagi

**Affiliations:** ^1^ Perinatal Center for Maternity and Neonates, Yokohama City University Medical Center Yokohama Japan; ^2^ Department of Obstetrics and Gynecology Yokohama City University Hospital Yokohama Japan

**Keywords:** dilutional coagulopathy, fibrinogen, fibrinolysis, obstetric disseminated intravascular coagulation (DIC), postpartum hemorrhage (PPH)

## Abstract

**Aim:**

In Japan, the diagnostic criteria for obstetric disseminated intravascular coagulation (DIC) were revised in 2024. This study aimed to evaluate the clinical utility of the revised obstetric DIC criteria, defined as the ability to identify patients at higher risk of severe maternal outcomes at presentation.

**Methods:**

This single‐center retrospective cohort study included women who delivered at ≥ 22 weeks of gestation and were transferred for primary postpartum hemorrhage (PPH) between April 2016 and March 2025. Women were compared according to obstetric DIC status and the presence of severe maternal outcomes to evaluate the ability of the revised criteria to identify patients with severe outcomes at presentation.

**Results:**

Among women transferred for primary PPH, 18% were diagnosed with obstetric DIC. Severe maternal outcomes occurred more frequently in the obstetric DIC group than in the non‐obstetric DIC group (55% vs. 24%; *p* = 0.002; relative risk, 2.27; 95% CI, 1.47–3.51). Fibrinogen levels were significantly lower in the severe group (163 mg/dL vs. 241 mg/dL, *p* < 0.001), whereas fibrin/fibrinogen degradation product (FDP) (85.6 μg/mL vs. 32.4 μg/mL, *p* = 0.61) and D‐dimer (11.2 μg/mL vs. 9.5 μg/mL, *p* = 0.083) levels showed no significant differences.

**Conclusions:**

Although the revised obstetric DIC criteria better reflect the underlying pathophysiology by incorporating hyperfibrinolysis, severe maternal outcomes were still observed in women without obstetric DIC, likely reflecting dilutional coagulopathy. These findings suggest that fibrinogen may serve as an indicator of maternal severity, complementing markers of enhanced fibrinolysis.

## 1 Introduction

Postpartum hemorrhage (PPH) is one of the leading causes of maternal mortality. A systematic analysis conducted by the World Health Organization (WHO) reported that hemorrhage accounts for 27% of maternal deaths, making it the most common cause worldwide [1]. In Japan, the maternal mortality ratio is approximately 4 per 100 000 births, and between 2020 and 2023, obstetric hemorrhage accounted for 15.8% of maternal deaths, second only to suicide (21.6%) [2]. Obstetric hemorrhage frequently leads to disseminated intravascular coagulation (DIC), referred to as obstetric DIC; however, no universally accepted diagnostic criteria currently exist.

In Japan, obstetric DIC scoring was first proposed by Maki et al. in 1985 [3, 4]. A major advantage of this scoring system was that it allowed early diagnosis and prompt initiation of treatment without waiting for laboratory coagulation results, relying instead on underlying conditions and clinical findings. However, the system is complex and involves diverse clinical conditions and symptoms. Its utilization rate remains relatively low, particularly in Japan, where approximately half of all deliveries occur in primary obstetric clinics [5].

More than 30 years have passed since the initial publication of the obstetric DIC score, and coagulation test results can now typically be obtained within 30–60 min. Accordingly, a simplified scoring system incorporating both laboratory findings reflecting coagulation and fibrinolytic abnormalities, as well as DIC pathophysiology, was developed. In 2022, tentative obstetric DIC criteria were introduced (Table 1) [6]. These criteria classified major underlying conditions (placental abruption, amniotic fluid embolism, and PPH with coagulopathy) and evaluated three factors—plasma fibrinogen, D‐dimer, and plasma fibrin/fibrinogen degradation products (FDP)—all of which require blood testing for diagnosis.

**TABLE 1:** Diagnostic criteria for obstetric DIC.

Tentative version [6]					
I. Underlying disease/pathology (select only one)	Points	II. Coagulability finding	Points	III. Fibrinolysis finding (select either a or b)	Points
a. Placental abruption	4	Fibrinogen (mg/dL)		a. FDP (μg/mL)	
b. Amniotic fluid embolism	4	≥ 300	0	< 30	0
c. Postpartum hemorrhage with coagulopathy	4	200–< 300	1	30–< 60	1
		150–< 200	2	≥ 60	2
		100–< 150^a^	3^a^	b. D‐dimer (μg/mL)	
		< 100^a^	4^a^	< 15	0
				15–< 25	1
				≥ 25	2
Obstetric DIC is diagnosed if the total of the three factors is ≥ 8 points.					

Final version [7]					
I. Underlying disease/pathology (select only one)	Points	II. Coagulability finding	Points	III. Fibrinolysis finding (select either a or b)	Points
a. Placental abruption	4	Fibrinogen (mg/dL)		a. FDP (μg/mL)	
b. Amniotic fluid embolism	4	≥ 300	0	< 30	0
c. Postpartum hemorrhage with coagulopathy	4	200–< 300	1	30–< 60	1
		150–< 200	2	≥ 60	2
		< 150^a^	3^a^	b. D‐dimer (μg/mL)	
				< 15	0
				15–< 25	1
				≥ 25	2

*Note:* Obstetric DIC is diagnosed if the total of the three factors is ≥ 8 points.

Abbreviations: DIC, disseminated intravascular coagulation; FDP, fibrin/fibrinogen degradation products.

^a^ Differences between the tentative version and the final version.

In 2024, these tentative criteria were updated, and the revised obstetric DIC criteria (final version) were published (Table 1) [7]. In the revised system, evidence of hyperfibrinolysis became a mandatory component for diagnosing obstetric DIC. Consequently, PPH cases with marked hypofibrinogenemia but without hyperfibrinolysis are no longer classified as obstetric DIC.

However, the revised criteria are diagnostic tools designed to assess the presence of DIC at a specific time point, and their clinical utility in identifying patients at higher risk of severe outcomes at presentation has not been fully clarified.

This study aimed to evaluate the clinical utility of the revised obstetric DIC criteria for identifying patients at higher risk of severe maternal outcomes at presentation.

## 2 Methods

This retrospective cohort study was conducted at Yokohama City University Medical Center, a tertiary perinatal center in Yokohama, Japan. Since 2016, our institution has implemented a specialized call system for managing transferred cases of PPH, referred to as the “PPH Call” system [8]. This in‐hospital alert is activated upon acceptance of a transfer request for a patient with PPH, mobilizing multidisciplinary teams to ensure rapid preparation and prompt transfusion. All transferred PPH patients were managed using this system, which enabled coordinated and immediate care upon arrival. This study included all patients with primary PPH at ≥ 22 weeks of gestation who were managed through the PPH Call system between April 1, 2016, and March 31, 2025. Patients were categorized into two groups according to the revised criteria: those diagnosed with obstetric DIC and those not diagnosed with obstetric DIC. The clinical utility of these criteria was evaluated based on their ability to identify patients with severe maternal outcomes at presentation. Additionally, patients were classified into severe and non‐severe maternal outcome groups, and maternal background characteristics and clinical findings were analyzed to explore factors associated with maternal severity.

### 2.1 Data Collection and Definitions

Patient data were obtained from our institutional database and medical records. The collected variables included maternal age, mode of delivery, parity, gestational age at delivery, fibrinogen level, D‐dimer or FDP level, diagnosis of obstetric DIC according to both the tentative and revised Japanese obstetric DIC criteria, estimated blood loss, and transfusion volume. Fibrinogen, D‐dimer, and FDP levels were measured in the central laboratory upon the patient's arrival at our institution. Primary PPH was defined as abnormal hemorrhage occurring within 24 h after delivery.

Based on underlying conditions and laboratory data at presentation, scoring was performed according to both the tentative and final Japanese obstetric DIC criteria, with a total score of ≥ 8 defined as obstetric DIC. When both D‐dimer and FDP levels were available, the higher of the two scores was used for the fibrinolytic component. Cases in which coagulation (fibrinogen) and fibrinolytic (D‐dimer or FDP) parameters were not obtained at the same time point at presentation, as well as those that had received transfusion at the referring hospital, were excluded. FDP data were frequently unavailable; therefore, D‐dimer was primarily used for the fibrinolytic assessment. Severe maternal outcomes were defined as the presence of at least one of the following: admission to the intensive care unit (ICU), endotracheal intubation in the emergency room (ER), Resuscitative Endovascular Balloon Occlusion of the Aorta (REBOA) placement in the ER, hysterectomy, acute kidney injury, cardiac arrest, or maternal death.

### 2.2 Statistical Analysis

Data are presented as medians [minimum–maximum] or numbers (percentage), excluding results below the detection limit, above the upper limit, and missing data. Statistical analyses were performed using JMP Student Edition 18.2.0 (SAS Institute Inc., Cary, NC, USA). The Mann–Whitney *U* test was used to compare median values, and Fisher's exact test was used to compare categorical variables. *p* < 0.05 were considered to indicate statistical significance for all analyses.

## 3 Results

**FIGURE 1:**
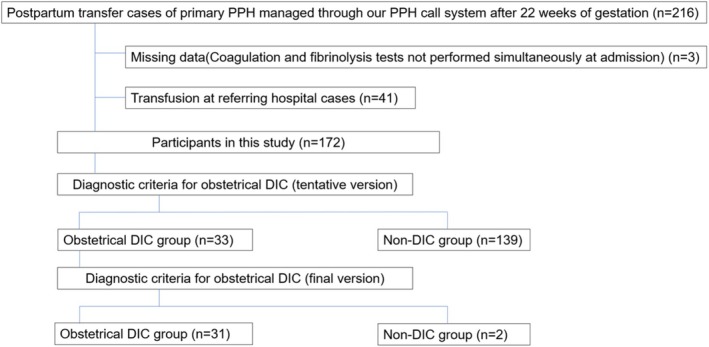
Flow diagram of study participants. Among 216 women transferred for primary PPH after 22 weeks of gestation, three were excluded due to missing simultaneous coagulation and fibrinolysis tests at admission, and 41 were excluded due to transfusion at the referring hospital. A total of 172 women were included in this study and classified according to the tentative and final versions of the criteria for obstetric disseminated intravascular coagulation (DIC). Based on the tentative criteria, 33 were categorized into the obstetric DIC group and 139 into the non‐DIC group. Based on the final criteria, 31 patients were categorized into the obstetric DIC group and two into the non‐DIC group.

A flow diagram of the study population is shown in Figure 1. During the study period, 216 women who delivered at ≥ 22 weeks of gestation were transferred to our institution for primary PPH and managed through the PPH Call system. After excluding three cases in which coagulation (fibrinogen) and fibrinolytic markers (D‐dimer or FDP) were not measured simultaneously at presentation, 213 cases remained. Of these, 41 women who had received transfusions prior to transfer were excluded, and 172 patients were included in the final analysis.

According to the tentative obstetric DIC criteria, 33 of the 172 women (19%) were diagnosed with obstetric DIC, whereas 31 women (18%) were diagnosed under the revised criteria. Among these 33 women, 31 (94%) were also classified as having obstetric DIC according to the revised criteria. The remaining two patients exhibited dilutional coagulopathy without hyperfibrinolysis (2/33, 6.1%).

Maternal background characteristics and laboratory findings at presentation are presented in Table 2. Maternal age, gestational age at delivery, nulliparity rate, and frequency of cesarean or instrumental delivery did not differ significantly between groups classified according to the revised criteria. Estimated blood loss was significantly higher in the obstetric DIC group (*p* < 0.001). Placental abruption and amniotic fluid embolism occurred only in the DIC group. Non‐clotting hemorrhage was also significantly more frequent in the DIC group. The proportions of hypofibrinogenemia cases (fibrinogen < 150 mg/dL) and hyperfibrinolysis cases (FDP ≥ 60 μg/mL or D‐dimer ≥ 25 μg/mL) were significantly higher in the DIC group. Fibrinogen and D‐dimer levels differed significantly between the groups (both *p* < 0.001). The proportion of cases with fibrinogen levels below the lower detection limit was 10 of 31 women (32%) in the DIC group and 1 of 141 women (0.7%) in the non‐DIC group (*p* < 0.001).

**TABLE 2:** Characteristics of participants at presentation according to the revised obstetric DIC criteria.

	Obstetric DIC (*n* = 31)	Non‐DIC (*n* = 141)	*p*
Maternal age (years)	35 [28–41]	34 [23–44]	0.063
Gestational weeks at delivery (weeks)	39.6 [35.9–41.7]	39.9 [36.0–42.0]	0.12
Primipara (%)	17 (55%)	82 (58%)	0.84
Cesarean section (%)	3 (9.7%)	15 (11%)	1.00
Forceps delivery (%)	4 (13%)	18 (13%)	1.00
Vacuum extraction (%)	5 (16%)	29 (21%)	0.80
Blood loss at delivery (g)	3490 [1758–24 285]	2616 [1001–8172]	< 0.001
Revised obstetric DIC score (points)	8 [8–9]	2 [0–7]	< 0.001
Underlying disease/pathology (points)	4 [4–4]	0 [0–4]	< 0.001
Placental abruption (4 points) (%)	1 (3.2%)	0 (0.0%)	0.18
Amniotic fluid embolism (4 points) (%)	1 (3.2%)	0 (0.0%)	0.18
Postpartum hemorrhage non‐clottable (4 points) (%)	30 (97%)	48 (34%)	< 0.001
Coagulability finding (points)	3 [2–3]	1 [0–3]	< 0.001
Fibrinogen < 150 mg/dL (3 points) (%)	25 (81%)	18 (13%)	< 0.001
Fibrinogen 150–< 200 mg/dL (2 points) (%)	6 (19%)	24 (17%)	0.79
Fibrinogen 200–< 300 mg/dL (1 point) (%)	0 (0.0%)	62 (44%)	< 0.001
Fibrinolysis finding (points)	1 [1–2]	0 [0–2]	< 0.001
FDP ≥ 60 μg/mL or D‐dimer ≥ 25 μg/mL (2 points) (%)	29 (94%)	15 (11%)	< 0.001
FDP ≥ 30 μg/mL or D‐dimer ≥ 15 μg/mL (1 point) (%)	2 (6.4%)	19 (13%)	0.37
Fibrinogen (mg/dL)	99.0 [53.0–175.0]	240.5 [60.0–501.0]	< 0.001
Fibrinogen < 50 mg/dL (%)	10 (32%)	1 (0.7%)	< 0.001
D‐dimer (μg/mL)	168.2 [22.2–860.7]	8.5 [2.2–69.2]	< 0.001
FDP (μg/mL)	434.9 [115.9–753.8]	32.3 [9.9–378.1]	0.051

*Note:* Data are shown as median [minimum–maximum] or number (percentage), excluding results below the detection limit, above the upper limit, and missing data. Fibrinogen: < detection limit (*n* = 11). D‐dimer: missing (*n* = 9), < detection limit (*n* = 1), and>upper limit (*n* = 1). FDP: missing (*n* = 157). At least one D‐dimer or FDP level was measured for all 172 women.

Abbreviations: DIC, disseminated intravascular coagulation; FDP, fibrin/fibrinogen degradation products.

Maternal outcomes are presented in Table 3. The frequencies and volumes of red blood cell (RBC), fresh frozen plasma (FFP), and platelet concentrate (PC) transfusions, as well as fibrinogen administration, were significantly higher in the obstetric DIC group. The proportion of patients with severe maternal outcomes, including ICU admission, endotracheal intubation in the ER, and hysterectomy, was also significantly higher in the DIC group.

**TABLE 3:** Maternal outcomes (blood transfusion, severe maternal outcomes).

	Obstetric DIC (*n* = 31)	Non‐DIC (*n* = 141)	*p*	Relative risk
RBCs (%)	31 (100%)	118 (84%)	0.016	—
Dosage (units)	12 [4–70]	6 [0–54]	< 0.001	—
FFP (%)	30 (97%)	79 (56%)	< 0.001	—
Dosage (units)	12 [0–74]	4 [0–68]	< 0.001	—
PCs (%)	15 (48%)	13 (9.2%)	< 0.001	—
Dosage (units)	0 [0–60]	0 [0–50]	< 0.001	—
Fibrinogen concentrates (%)	31 (100%)	61 (43%)	< 0.001	—
Dosage (g)	6 [3–15]	0 [0–10]	< 0.001	—
Severe maternal outcomes (%)	17 (55%)	34 (24%)	0.002	2.27 (95% CI 1.47–3.51)
ICU admission (%)	15 (48%)	28 (20%)	0.002	2.44 (95% CI 1.49–3.99)
Intubation in the ER (%)	11 (36%)	10 (7.1%)	< 0.001	5.00 (95% CI 2.33–10.7)
REBOA placement in the ER (%)	6 (19%)	10 (7.1%)	0.044	2.73 (95% CI 1.07–6.95)
Hysterectomy (%)	7 (22%)	8 (5.7%)	0.007	3.98 (95% CI 1.56–10.2)
Acute kidney injury (%)	2 (6.5%)	2 (1.4%)	0.15	4.55 (95% CI 0.67–31.1)
Cardiac arrest (%)	3 (9.7%)	2 (1.4%)	0.041	6.82 (95% CI 1.19–39.1)
Maternal death (%)	0 (0.0%)	0 (0.0%)	—	—

Abbreviations: DIC, disseminated intravascular coagulation; ER, emergency room; FDP, fibrin/fibrinogen degradation products; FFP, fresh frozen plasma; ICU, intensive care unit; PCs, platelet concentrates; RBCs, red cell concentrates; REBOA, resuscitative endovascular balloon occlusion of the aorta.

Severe maternal outcomes were also observed in women classified as non‐DIC according to the revised criteria. Notably, two patients with severe hypofibrinogenemia but without hyperfibrinolysis (dilutional coagulopathy) met the criteria for obstetric DIC under the tentative criteria but not under the revised criteria; both experienced severe maternal outcomes. Representative cases are described below.

One case involved a massive hemorrhage after a forceps delivery with retained placenta and uterine inversion (blood loss at the referring hospital, 2400 g). Upon arrival, laboratory tests showed fibrinogen and D‐dimer levels of 60 mg/dL and 14.9 μg/mL, respectively. Total blood loss was 6000 g, and the patient required manual placental removal and massive transfusion (fibrinogen, 3 g; RBCs, 12 U; FFP, 6 U; PCs, 20 U). She required ICU admission and was classified as having a severe maternal outcome.

Another patient had a massive hemorrhage due to uterine dehiscence and retained placenta after vaginal delivery following oxytocin augmentation (blood loss at the referring hospital, 2600 g). On arrival, the fibrinogen level was < 50 mg/dL, and the D‐dimer level was 5.1 μg/mL. Total blood loss reached 6647 g, requiring transcatheter arterial embolization (TAE) and massive transfusion (fibrinogen, 3 g; RBCs, 24 U; FFP, 22 U; PCs, 20 U). She underwent emergency intubation in the ER and ICU admission and was classified as having a severe maternal outcome. Subsequently, a laparoscopic hysterectomy was performed for the retained placenta.

A comparison of patients with and without severe maternal outcomes is shown in Table 4. Fibrinogen levels at presentation were significantly lower in the severe group than in the non‐severe group (163 vs. 241 mg/dL, *p* < 0.001). In contrast, no significant differences were observed in FDP (85.6 μg/mL vs. 32.4 μg/mL, *p* = 0.61) or D‐dimer levels (11.2 μg/mL vs. 9.5 μg/mL, *p* = 0.083).

**TABLE 4:** Comparison of women with and without severe maternal outcomes.

	Severe maternal outcomes (*n* = 51)	Non‐severe maternal outcomes (*n* = 121)	*p*
Maternal age (years)	34 [24–44]	34 [23–42]	0.48
Gestational weeks at delivery (weeks)	39.7 [35.9–41.7]	39.9 [36–42]	0.89
Primipara (%)	30 (59%)	69 (57%)	0.87
Cesarean section (%)	4 (7.8%)	14 (12%)	0.59
Forceps delivery (%)	8 (16%)	14 (12%)	0.46
Vacuum extraction (%)	11 (22%)	23 (19%)	0.68
Blood loss at delivery (g)	3920 [1697–24 285]	2380 [1001–6766]	< 0.001
Fibrinogen (mg/dL)	163 [53–422]	241 [71–501]	< 0.001
Fibrinogen < 50 mg/dL (%)	9 (18%)	2 (1.7%)	< 0.001
Fibrinogen < 100 mg/dL (%)	16 (31%)	8 (6.6%)	< 0.001
Fibrinogen < 150 mg/dL (%)	28 (55%)	15 (12%)	< 0.001
Fibrinogen < 200 mg/dL (%)	35 (69%)	38 (31%)	< 0.001
D‐dimer (μg/mL)	11.2 [2.8–860.7]	9.5 [2.2–524.0]	0.083
D‐dimer < 15 μg/mL (%)	29 (59%, *n* = 49)	77 (68%, *n* = 114)	0.37
D‐dimer ≥ 15 μg/mL (%)	20 (41%, *n* = 49)	37 (32%, *n* = 114)	0.37
D‐dimer ≥ 25 μg/mL (%)	17 (35%, *n* = 49)	24 (21%, *n* = 114)	0.077
FDP (μg/mL)	85.6 [19.5–115.9]	32.4 [9.9–753.8]	0.61
RBCs (%)	51 (100%)	98 (81%)	< 0.001
Dosage (units)	14 [4–70]	4 [0–26]	< 0.001
FFP (%)	51 (100%)	58 (48%)	< 0.001
Dosage (units)	12 [2–74]	0 [0–22]	< 0.001
PCs (%)	24 (47%)	4 (3.3%)	< 0.001
Dosage (units)	0 [0–60]	0 [0–50]	< 0.001
Fibrinogen concentrates (%)	46 (90%)	46 (38%)	< 0.001
Dosage (g)	3 [3–15]	0 [0–9]	< 0.001

*Note:* Data are shown as median [minimum–maximum] or number (percentage), excluding results below the detection limit, above the upper limit, and missing data. Fibrinogen: < detection limit (*n* = 11). D‐dimer: missing (*n* = 9), < detection limit (*n* = 1), and > upper limit (*n* = 1). FDP: missing (*n* = 157). At least one D‐dimer or FDP level was measured for all 172 women.

Abbreviations: FDP, fibrin/fibrinogen degradation products; FFP, fresh frozen plasma; RBCs, red cell concentrates, PCs, platelet concentrates.

## 4 Discussion

This study yielded several important findings. First, severe maternal outcomes were more frequently observed among women diagnosed with obstetric DIC at presentation according to the revised criteria than among those without obstetric DIC. Second, some cases characterized by severe hypofibrinogenemia without hyperfibrinolysis (dilutional coagulopathy), which were classified as obstetric DIC under the tentative criteria but not under the revised criteria, were also observed among patients with severe maternal outcomes. Finally, when comparing women with and without severe maternal outcomes, fibrinogen levels were more closely associated with maternal severity than fibrinolytic markers. These findings may reflect differences in the underlying pathophysiology, including the presence of dilutional coagulopathy without hyperfibrinolysis, and suggest that fibrinogen may serve as an early indicator of maternal severity, while also highlighting both the clinical utility and the scope of application of the revised criteria.

First, the proportion of severe maternal outcomes was significantly higher among women diagnosed with obstetric DIC according to the revised criteria than among those without obstetric DIC. Obstetric DIC is generally classified into enhanced–fibrinolytic‐type and suppressed–fibrinolytic‐type DIC [9], with most falling into the enhanced–fibrinolytic category. Because the revised criteria require evidence of hyperfibrinolysis, they better capture the characteristic features of enhanced–fibrinolytic‐type DIC observed in placental abruption, amniotic fluid embolism, and non‐clotting hemorrhage. In contrast, sepsis, including fulminant group A streptococcal infection, often presents with features of suppressed–fibrinolytic‐type DIC, although overlap with enhanced–fibrinolytic features may occur; therefore, such cases are not the primary target of the revised obstetric DIC criteria. In June 2024, the Japan Society of Obstetrical, Gynecological and Neonatal Hematology (JSOGNH) recommended that obstetric DIC induced by a hematopoietic disorder should be diagnosed according to the DIC scoring system for critical illness of the Japanese Association for Acute Medicine [10] instead of the new Japanese obstetric DIC criteria [7]. The revised criteria were developed based on these pathophysiological considerations, and our findings support their validity in identifying enhanced–fibrinolytic‐type DIC.

Second, some women with severe hypofibrinogenemia without evidence of hyperfibrinolysis, who were not diagnosed with obstetric DIC under the revised criteria, were observed among patients with severe maternal outcomes. These cases likely represent dilutional coagulopathy, a distinct pathophysiological entity from obstetric DIC, which may occur during massive hemorrhage with fluid resuscitation and transfusion [11]. In dilutional coagulopathy, coagulation factors decrease, whereas fibrinolytic markers such as FDP and D‐dimer may remain within normal ranges; therefore, such cases may not fulfill the diagnostic definition of obstetric DIC by design. Even in such cases, patients may still require intensive management, including massive transfusion, intubation, and ICU care. Early transfer to tertiary care centers remains important for timely and appropriate management. An important consideration is whether cases presenting with hypofibrinogenemia without hyperfibrinolysis subsequently developed elevations in FDP or D‐dimer during their clinical course. If hyperfibrinolysis emerged later, these cases may represent early‐stage coagulopathy progressing toward overt DIC, and the revised criteria may appropriately reflect the disease stage at presentation. However, although laboratory data at presentation were available for all cases, follow‐up measurements were not standardized in terms of timing, parameters, or clinical interventions, precluding systematic evaluation of temporal changes. This should be considered a limitation.

Finally, fibrinogen levels at presentation were significantly lower in the severe maternal outcome group than in the non‐severe group, whereas FDP and D‐dimer levels did not differ significantly. These findings suggest that fibrinogen may be more closely associated with maternal severity than fibrinolytic markers, consistent with previous reports identifying hypofibrinogenemia as an early indicator of severe PPH [12]. Dilutional coagulopathy does not consistently exhibit hyperfibrinolysis, unlike consumptive coagulopathy, but may still require multidisciplinary management similar to obstetric DIC. Some authors have suggested that, in the setting of abnormal obstetric hemorrhage, avoiding excessive crystalloid infusion or RBC transfusion alone and earlier initiation of coagulation factor replacement, such as FFP, may help reduce the risk of dilutional coagulopathy [13].

These findings indicate that while the revised criteria reliably identify enhanced–fibrinolytic‐type DIC, they may not be designed to capture the clinical severity of dilutional coagulopathy, which represents a distinct pathophysiological entity. In clinical practice, fibrinogen measurement and the revised obstetric DIC criteria should be considered complementary tools, as emphasized in the Japanese Clinical Practice Guide for Critical Obstetrical Hemorrhage 2022 [14], in which fibrinogen is incorporated as a key parameter for early decision‐making. While fibrinogen levels provide a rapid and practical indicator for assessing hemorrhage severity and guiding timely intervention, the revised criteria offer a structured framework for diagnosing obstetric DIC based on underlying pathophysiology. Therefore, these approaches should be used in combination rather than in isolation to optimize clinical decision‐making. In addition, because the minimum concentration required for effective hemostasis varies across coagulation factors, and fibrinogen has the highest threshold [15], it is often the first factor to reach critically low levels during massive hemorrhage. Several studies have suggested that the risk of severe obstetric hemorrhage is closely related to fibrinogen levels [16], underscoring the importance of rapid fibrinogen measurement and early supplementation. Additionally, recent advances in point‐of‐care testing allow rapid assessment of fibrinogen levels [17], contributing to earlier and more effective hemorrhage management.

This study has several limitations. First, it was a single‐center retrospective study with a limited sample size; therefore, caution is required when generalizing the findings. Second, although the management strategy remained largely consistent over the nine‐year study period, changes in staffing, including personnel turnover, may not have been fully captured. Third, due to the retrospective design, temporal changes in fibrinolytic markers could not be fully evaluated. Fourth, tranexamic acid was used at our institution according to the clinical situation, and its potential impact on maternal outcomes could not be excluded. Nevertheless, all cases were comprehensively evaluated at a tertiary perinatal center, and the data were considered accurate and reliable.

In conclusion, the revised obstetric DIC criteria are useful diagnostic tools for identifying enhanced–fibrinolytic‐type DIC at presentation. Because they are based on the presence of hyperfibrinolysis, they may not classify cases of hypofibrinogenemia without hyperfibrinolysis, such as dilutional coagulopathy, which represents a distinct pathophysiological entity. Among the evaluated parameters, fibrinogen levels may be more closely associated with maternal severity, and hypofibrinogenemia may serve as an early indicator of clinical deterioration. In clinical practice, fibrinogen measurement and the revised obstetric DIC criteria should be considered complementary tools, and clinical assessment incorporating both approaches is important for timely and appropriate management. Future multicenter prospective studies are warranted to further refine severity assessment and therapeutic algorithms for obstetric hemorrhage.

## Author Contributions

**Soichiro Obata:** conceptualization, methodology, validation, formal analysis, writing – original draft, writing – review and editing, visualization. **Sayuri Nakanishi:** writing – review and editing. **Etsuko Miyagi:** writing – review and editing. **Yutaro Takahashi:** conceptualization, investigation, methodology, validation, formal analysis, writing – original draft, visualization. **Shigeru Aoki:** conceptualization, methodology, writing – review and editing, supervision, project administration. **Ryosuke Shindo:** writing – review and editing.

## Funding

The authors have nothing to report.

## Disclosure

A preliminary version of this study was presented at the 77th Annual Meeting of the Japan Society of Obstetrics and Gynecology held in Okayama, Japan, in May 2025. Drs. Shigeru Aoki and Soichiro Obata serve on the editorial board of the *Journal of Obstetrics and Gynaecology Research* and are co‐authors of this manuscript. To prevent any potential conflicts of interest, they had no involvement in the editorial handling or peer review of this submission.

## Ethics Statement

This study was conducted in accordance with the principles of the Declaration of Helsinki and was approved by the Ethics Committee and Institutional Review Board of Yokohama City University Medical Center (approval number: 2025‐043).

## Consent

As this was a retrospective analysis using data extracted from medical records, written informed consent was not required. In accordance with the Ethics Committee's approval, the details of the study were made publicly available on the hospital website to allow patients the opportunity to opt out.

## Conflicts of Interest

The authors declare no conflicts of interest.

## Data Availability

The data supporting the findings of this study were obtained from medical records collected at Yokohama City University Medical Center. Although all data were deidentified prior to analysis, they originate from sensitive clinical information and cannot be made publicly available owing to ethical and privacy restrictions imposed by the Institutional Review Board. De‐identified aggregate data may be made available by the corresponding author upon reasonable request and with approval from the Ethics Committee of Yokohama City University Medical Center.
